# Limits of the Surgeon’s Responsibility

**Published:** 1877-03

**Authors:** Francis H. Milligan

**Affiliations:** Wabashaw, Minn.


### LIMITS OF THE SURGEON'S RESPONSIBILITY.

BY FRANCIS H. MILLIGAN, M. D., WABASHAW, MINN.

*Read before the State Medical Society, February* 1st, 1877.

The responsibility of the medical man, more particularly the surgeon, has been, and is now, the source of a great deal of medico-legal discussion.

In this country and in England the great weight of the authorities, is that no distinction exists between regular and irregular physicians and surgeons.*

First. If the defendant acted honestly, and used his best skill to cure, and it does not appear that he thrust himself in the place of a competent person, it makes no difference whether he was at the time a regular physician and surgeon or not.

Second. To constitute guilt, gross ignorance or negligence must be proved.

Third. A defendant, who, with competent knowledge, makes a mistake in a remedy, is not answerable; but it is otherwise when a violent remedy, shown to have occasioned death, is administered by a person grossly ignorant, but with average capacity, in which case malice is presumed, in the same way that it is presumed when a man, *compos mentis*, lets loose a mad bull into a thoroughfare, or casts down a log of wood on a crowd.

Fourth. When competent medical aid can be had, the application of violent remedies by an ignorant person, though with the best motives, involves him in a criminal responsibility.

Fifth. Express malice, or an intent to commit a personal or social wrong, make the practitioner criminally responsible in all cases of mischief.

There is a growing opinion in the community, that the surgeon should be held responsible for all the failures that occur in his practice. If a deformity results from a fracture, or an operation proves a failure, the surgeon is not only blamed by the community at large, but, unfortunately, his own brethren are too often ready to lend their aid to the general clamor. Therefore, at least two-thirds of all the difficulties resulting from malpractice suits are the result of words spoken in an unguarded moment by some rival practitioner. This is not the case with the practitioner of medicine, for however badly a case of disease may have been treated, few, if any, physicians are blamed for the results. On the other

*See Wharton & Stille, Ed. 1860, Sec. 1253.

hand, let a surgeon cure a fracture with deformity, or reduce a dislocation, or fail to cure a diseased joint, and he is too often blamed for the results, over which he had really no more control than the physician over the disease treated by him.

Years ago, in civil practice, I attended a case of gunshot wound of the left lung, penetrating the cavity of the thorax, the ball being extracted by me from near the spinal column, on a line corresponding to the lower border of the left scapula. This case remained under my care for about three years, the wound of entrance never healing. The patient, being desirous of having the opinion of some more experienced surgeon, went to St. Louis, and consulted the late Professor Charles A. Pope. After having examined the case thoroughly, Dr. Pope remarked, in that kind and gentle manner which was so peculiar to him, "My man, it is too late; I can do nothing for you." Now, this remark caused my patient to return to his home with the idea that I was responsible for his not being cured. I immediately wrote to Dr. Pope, and received an answer, in due course of time, expressing the following language: "All was done by you, in the case, that could have been done by any man (good or bad surgeon). The result would probably have been fatal under any treatment, and you, my friend, are not to blame or responsible, for the result. The remarks uttered by me to Mr. W. were intended to mean far different from what he construed them, and is another instance to show that we can not be too guarded in our language to the patients of others."

Public opinion, however, will very often err in regard to the success of a surgeon. Operations of the most simple nature get into the hands of the reporters of the secular press, and honor is too often bestowed where none should be given.

Again, our successes are heralded far and near, and our failures are too often hidden from the public gaze and curiosity. Surgeons, as a class, are subject to all the frailties of other men, and we do not like to hear of our failures, but, in reality, enjoy the public esteem too often dishonestly obtained at the expense of our professional honor. It does rest upon us all to consider these things very carefully.* We are all the more bound to consider them honestly, and, if need be, with self-reproach, because these calamities are not such as the public can judge of; they are not instances of those negligences and carelessness which can be punished legally and openly.

All men are liable to error. Men of the largest experience, and of undoubted ability, often make very serious mistakes; for this reason it has always been my custom to request the opinion of my professional brethren, and thus divide the responsibility in case of failure.

We can not pass over the remarks of one of England's most successful surgeons.† I venture to say that there is no surgeon in large practice, no surgeon to a large hospital, who has not once or more, in the course of his life, shortened patient's lives when he was making attempts either to prolong them or make them happier. And this, you will observe, is not merely the case with capital operations. When a patient submits to a large operation, it is always for the remedy of something that will render his life either very miserable or very short; and to escape so great distress, it is quite fair that a man should run great risk of his life.

Calamities in surgery may come of things which nothing short of omniscience could have detected beforehand.

Tetanus may ensue, after a minor operairon. We have no power to avert that, or even in the smallest degree, to apprehend its approach. These, and other causes of the kind, and still more, the negligence of patients, would diminish, by a considerable number, the list of calamities for which we seem to be blameworthy.

There is a limit to the responsibility. Notwithstanding the greatest care and attention, deformities will sometimes occur, and a surgeon who has been in active business for a period of years, if honest, will admit that some of his cases resulted badly. We know that more than two-thirds of the malprac-

*Paget's "Clinical Lectures," p. 51.

†Paget's "Clinical Lectures and Essays," p. 53.

tice cases are the result of fractures, and the deformity therefrom. If under the best care these deformities result, as a natural consequence, under the care of our country surgeons, who are but poorly provided with surgical appliances, they are to be expected oftener. In hospital practice or in our large cities better cures are made in fractures, not because the attending surgeon possesses more ability than his rural neighbor, but because he has greater access to his patient, seeing him daily, or if necessary, twice daily. This would be impossible in our rural districts, and hence we must all admit that the country surgeon meets with more serious results than the surgeons of our large cities.

An American surgeon* who at home and abroad justly stands in the front ranks of his profession in the "green room," has been in the habit of remarking that a surgeon in Wisconsin should know as much as one in Philadelphia, and insists that no man should take the responsibility of treating a fracture that he could not see daily.

The common law expects a surgeon to see his case as often as the circumstances will admit. Neglect is a hard subject to go before a jury. It is right that it should be so. We will go as far as any man to punish neglect and ignorance, on the part of a surgeon, but this general and vulgar clamor about a surgeon's being rich, and ought to be made to pay for the shortening of the limb of some pauper case which has fallen into the hands of some ambitious attorney, is unworthy the jurisprudence of the age, and ought to be done away with.

The habit pursued by the eminent Dr. E. B. Walcott, of Milwaukee, should be imitated by us all. When he is consulted by parties wishing an opinion to establish a case of malpractice, he examines the case thoroughly, takes notes, and invariably remarks to the parties, "what I know of this case, sir, I will tell to the court and jury." How much better is this remark than the one so often given; "a bad leg sir, *very bad*." "I have no such arm as this in my practice."—*Med. & Surg. Reporter*.

*Professor S. D. Gross.

